# Microbial responses to multi-factor climate change: effects on soil enzymes

**DOI:** 10.3389/fmicb.2013.00146

**Published:** 2013-06-11

**Authors:** J. Megan Steinweg, Jeffrey S. Dukes, Eldor A. Paul, Matthew D. Wallenstein

**Affiliations:** ^1^Natural Resource Ecology Laboratory, Colorado State UniversityFort Collins, CO, USA; ^2^Graduate Degree Program in Ecology, Colorado State UniversityFort Collins, CO, USA; ^3^Department of Forestry and Natural Resources, Purdue UniversityWest Lafayette, IN, USA; ^4^Department of Biological Sciences, Purdue UniversityWest Lafayette, IN, USA; ^5^Department of Soil and Crop Sciences, Colorado State UniversityFort Collins, CO, USA; ^6^Department of Ecosystem Science and Sustainability, Colorado State UniversityFort Collins, CO, USA

**Keywords:** enzymes, carbon, nitrogen, precipitation, temperature, decomposition, microbial biomass

## Abstract

The activities of extracellular enzymes, the proximate agents of decomposition in soils, are known to depend strongly on temperature, but less is known about how they respond to changes in precipitation patterns, and the interaction of these two components of climate change. Both enzyme production and turnover can be affected by changes in temperature and soil moisture, thus it is difficult to predict how enzyme pool size may respond to altered climate. Soils from the Boston-Area Climate Experiment (BACE), which is located in an old field (on abandoned farmland), were used to examine how climate variables affect enzyme activities and microbial biomass carbon (MBC) in different seasons and in soils exposed to a combination of three levels of precipitation treatments (ambient, 150% of ambient during growing season, and 50% of ambient year-round) and four levels of warming treatments (unwarmed to ~4°C above ambient) over the course of a year. Warming, precipitation and season had very little effect on potential enzyme activity. Most models assume that enzyme dynamics follow microbial biomass, because enzyme production should be directly controlled by the size and activity of microbial biomass. We observed differences among seasons and treatments in mass-specific potential enzyme activity, suggesting that this assumption is invalid. In June 2009, mass-specific potential enzyme activity, using chloroform fumigation-extraction MBC, increased with temperature, peaking under medium warming and then declining under the highest warming. This finding suggests that either enzyme production increased with temperature or turnover rates decreased. Increased maintenance costs associated with warming may have resulted in increased mass-specific enzyme activities due to increased nutrient demand. Our research suggests that allocation of resources to enzyme production could be affected by climate-induced changes in microbial efficiency and maintenance costs.

## Introduction

The rate at which soil organic matter (SOM) is decomposed is strongly affected by temperature and moisture, and thus should be sensitive to climate change (Davidson et al., 1998; Schimel and Gulledge, 1998). While heterotrophic respiration is widely used as a proxy for decomposition, the relationship between abiotic drivers and decomposition rates is driven by a series of underlying microbially mediated processes (Ekschmitt et al., 2005). For example, enzymatic depolymerization of SOM controls the rate at which assimilable dissolved organic matter (DOM) is produced (Conant et al., 2011), and has been hypothesized to be the rate-limiting step in decomposition (Schimel and Bennett, 2004; Bengtson and Bengtsson, 2007). Thus, it is important to examine the response of enzyme activities to climate change in order to improve our ability to predict carbon fluxes under future climate regimes.

The rate of *in situ* enzyme activity is directly responsive to temperature and moisture (Trasar-Cepeda et al., 2007; Allison and Treseder, 2008; Wallenstein and Weintraub, 2008; Steinweg et al., 2012) but is also controlled by enzyme pool size. Enzyme pool size is controlled by the rate at which enzymes are produced by microbes relative to the rate at which they are degraded in the environment. Both enzyme production and turnover rates may be affected by temperature and moisture, and thus may vary seasonally and be affected by climate change.

What controls enzyme production by microbes? The production of enzymes incurs a cost to microbes in terms of both energy and nutrients. Thus, the production of enzymes should be governed by the economics of the amount of resources gained for each enzyme produced (Allison et al., 2011). To maintain the stoichiometry of their biomass (driven by the fixed stoichiometry of cellular components) (Cleveland and Liptzin, 2007), microbes produce enzymes targeting specific compounds that are rich in either carbon (C), nitrogen (N) or phosphorus (P) (Sinsabaugh et al., 2008, 2009). However, enzyme production declines for many substrates when substrate concentration is low (German et al., 2011).

Temperature and moisture can affect both the overall rate of enzyme production as well as the relative rate of production of different enzymes due to effects on enzyme efficiency, substrate availability, and microbial efficiency. Thus, changes in the soil microclimate, whether they occur within hours, weeks, seasonally, or over decades in response to climate change, will affect enzyme pool sizes. In response to increased activity of the extant enzyme pool as soil temperatures increase, given available substrate, microbes may allocate fewer resources to enzyme production if microbial biomass remains unchanged (Allison and Vitousek, 2005). Several studies have found that N-degrading enzymes have lower temperature sensitivities than C-degrading enzymes (Wallenstein et al., 2009, 2012; Stone et al., 2012). This could result in increasing N limitation as soils warm, spurring microbes to increase the production of N-degrading enzymes and decrease the production of C-degrading enzymes. Soil moisture affects the diffusion of substrates, enzymes and the products of enzyme activity, and thus drought conditions could impose diffusion limitations on enzymes and substrates (Stark and Firestone, 1995; Allison, 2005). In oxic soils, drought could decrease enzyme production as biomass declines, or increase production to satisfy nutrient requirements of the biomass (Allison and Vitousek, 2005; Sardans and Peñuelas, 2005; Sowerby et al., 2005).

Our objective was to assess the response of hydrolytic enzyme activities to a multi-factor climate change experiment. To separate the influences of soil warming and moisture on enzyme activity from seasonal effects, we measured the activity and stoichiometry of six enzymes involved in C, N, and P cycling four times over the course of a year, in soils from the Boston-Area Climate Experiment (BACE). The BACE is a multifactorial climate change manipulation in an old-field ecosystem, providing three levels of precipitation and four levels of warming. Because of the multifactorial design, the BACE allowed us to compare twelve different climate years simultaneously during the 1 year of soil sampling. Potential enzyme activity is a metric for soil microbial function response to disturbance (Henry et al., 2005) and indicates shifts in metabolic requirements (Caldwell, 2005). We hypothesized that climate affects enzyme activity by altering microbial biomass and through abiotic controls on enzyme turnover and stabilization. We predicted that: (1) drought would reduce microbial biomass, decreasing potential enzyme activity, (2) warming in the field would decrease potential enzyme activity measured in the laboratory, because enzymes produced at higher temperatures would have higher reaction efficiency, resulting in decreased microbial enzyme production rates, and (3) N and P enzyme activities would be greater in the growing season compared to the winter due to increased C availability.

## Materials and methods

### Study site

The BACE is located in an old field in Waltham, Massachusetts at the University of Massachusetts' Suburban Experiment Station (42° 23′ 3″N, 71° 12′ 52″ W; “old fields” are typically abandoned agricultural fields dominated by perennial grasses and forbs; they are kept from returning to their pre-agricultural forested state by regular mowing or grazing). Mean annual precipitation and temperature in the area are 1194 mm yr^−1^ and 9.5°C (Hoeppner and Dukes, 2012). The soil is a mesic Typic Dystrudept, and the upper 30 cm is loam (45% sand, 46% silt, and 9% clay), with an average pH of 5.5. The site, a former apple orchard, has harbored old-field vegetation for more than 40 years. Recent surveys identified 42 grass and forb species, most of which have been introduced (Hoeppner and Dukes, 2012).

### Field experimental design

The BACE exposed 36 square, 4 m^2^ plots to three precipitation treatments and four warming levels in a full-factorial design, with three replicates of each treatment. The precipitation treatments included an “ambient” control, a “wet” treatment that received a 50% increase in precipitation during the growing season only, and a “drought” treatment in which 50% of ambient precipitation was excluded across all seasons. These treatments were chosen such that a year with average precipitation would result in “wet” and “drought” treatments that fell within the extremes of a 75-year historical record for the area. Above the drought plots, clear partial roofs excluded half of incoming precipitation, and this water was immediately diverted to wet plots from May to October. The roofs continued to function from November to April, but during these colder months diverted water was not added to the wet plots. Drought treatments began in January 2007, and wet treatments began in June 2008.

The warming treatments (unwarmed ambient, low, medium, and high) were implemented such that warming of the canopy in the high treatment was limited to a maximum of 4°C. This temperature limit was determined by logistical and financial constraints. Warming was achieved using ceramic infrared heaters, which were mounted 1 m above each corner of each plot. An unwarmed treatment had four dummy heaters (providing similar shade as heaters, but no warming), and individual heaters above the low, medium, and high treatments were rated at 200, 600, and 1000 W, respectively. Warming treatments were nested within precipitation treatments; within each area receiving a given precipitation treatment, a group of four plots was arranged linearly, from unwarmed to high. Canopy temperature was monitored every 10 s in the unwarmed and high plots in each group, using infrared radiometers (IRR-PN; Apogee Instruments, Logan, UT, USA). All heaters in each group of four plots were controlled by the same circuit, and the system was programmed to adjust power to the circuit to maintain a target difference of 4°C between the “high” and “unwarmed” plots in each group. Warming treatments began on July 1, 2008.

Soil moisture was measured weekly during the non-freezing months, usually beginning in April and ending in December, using time-domain reflectometry (waveguides were installed across 0–10 and 0–30 cm depths). Dataloggers recorded soil temperature near the center of each plot every 30 min throughout the year, as measured by linear temperature sensors positioned at 2 and 10 cm depths. Field measurements of heterotrophic soil respiration were taken using a LI-COR 6400-09 soil CO_2_ flux chamber attached to a 6400 portable photosynthetic system. Once a month, CO_2_ flux was measured within a 25 cm diameter PVC collar installed in each plot. Collars extended to 30 cm depth, and had been installed in November 2007. All plants were removed from the collar shortly after installation, and collars were subsequently covered with a weed-blocking cloth to prevent new plants from colonizing the soil [for details see Suseela et al. (2012)].

### Soil sampling and processing

Soils were first collected from all plots in June 2008, 1 year after precipitation manipulations began, but before the start of the warming treatments. Additionally, soil samples were taken three times (August 2008, January 2009, and June 2009) following the initiation of the warming treatment. Three cores (5 cm diameter) were collected from each plot at 0–5 and 5–15 cm depths. Soils were packaged on ice and shipped to the laboratory overnight, where the cores from each plot were sieved (2 mm), picked free of rocks and roots, homogenized and frozen at −10°C until analysis.

### Soil characterization

Subsamples from each plot were taken for determination of percent soil moisture, pH, and total C and N concentrations. Soil moisture was determined after field-moist soils were weighed and dried for 48 h at 60°C and then reweighed. Soil pH was determined using the supernatant of soil mixed with water (1:5 by volume). Soil subsamples were dried at 60°C and ground to measure total C and N concentrations on a LECO CHN-1000 autoanalyzer (LECO Corporation, St. Joseph, MI, USA).

### Microbial biomass

Substrate-induced respiration (SIR) and chloroform fumigation extraction (CFE) were used to estimate microbial biomass carbon (MBC) (Anderson and Domsch, 1978; Vance et al., 1987). SIR-MBC is an estimation of the active microbial biomass whereas CFE-MBC is an estimation of the total microbial biomass.

#### SIR-MBC

SIR-MBC was measured using a deep-well microplate-based technique called MicroResp™ (Aberdeen, UK) (Campbell et al., 2003). Soils from all sampling dates were removed from the freezer and a 20 g subsample was thawed to about 20°C within 3 h. Since soil moisture varied by date, we brought all soils to 55% water holding capacity through wetting or drying, for optimum microbial activity and to eliminate substrate diffusion constraints. The August 2008 and June 2009 samples were initially below 55% water holding capacity, so after thawing, all August and June samples had water added. Samples were then covered for 1 h, homogenized and added to wells in the 96-well deep-well plates. For the January 2009 sampling, all samples were over 55% water holding capacity. In this case, 20 g subsamples were dried to 55% water holding capacity at 4°C, over 6–36 h. Following drying, the January 2009 samples were homogenized and weighed into 96-well deep-well plates. Three wells on a plate were used per sample, with about 0.2–0.3 g of moist soil added to each well, using the MicroResp manufacturer's protocol. After samples were added to the deep-well plate, they were covered with sealing film and placed at 4°C for about 18 h prior to addition of glucose.

Following the 18 h incubation at 4°C, 25 μl of 1 M glucose solution was added (this concentration had been determined to saturate demand in preliminary assays), and samples were then incubated at 25°C for 6 h. The CO_2_ indicator plates were read on a Tecan Infinite M500 microplate reader at 625 nm prior to being placed on deep-well plates. The indicator plate and deep-well plate were attached to one another using the MicroResp apparatus and allowed to incubate. Following the 6 h incubation the indicator plates were removed from the deep-well plates and read again on the Tecan microplate reader at 625 nm.

Indicator plates (containing cresol red, sodium bicarbonate and potassium chloride) were made 1 week in advance of the assay according to the manufacturer's guidelines. Standard curves were generated by incubating indicator plates in jars filled with known concentrations of CO_2_. The amount of CO_2_ produced from the water addition wells was subtracted from the respiration in the glucose addition wells to account for stimulation of respiration due to changes in soil water content. MBC was calculated from respiration produced from the glucose amended wells at 25°C and using the following equation from Anderson and Domsch (1978):

$$\text{mg MBC }100{\text{g}}^{-1}\text{soil}=40.04y+0.37$$

where *y* is the amount of CO_2_ produced under glucose amendment.

#### CFE-MBC

CFE-MBC was measured using the method of Vance et al. (1987). Briefly, 10 g of field-moist soil from each plot was thawed and placed in a fumigation chamber and fumigated over the course of 5 days with chloroform. Following fumigation, the soils were shaken with 40 mL of 0.5 M K_2_SO_4_ for 2 h and then filtered through a Whatman 1 filter. Additionally, another 10 g sample from each plot was shaken for 2 h with 0.5 M K2SO4 and then filtered through a Whatman 1 filter. The filtrates were stored frozen until analysis. The organic carbon in the filtrates from both procedures was measured on a Shimadzu TOC analyzer (Shimadzu Scientific Instruments, Columbia, MD, USA). The fumigated sample contained dissolved organic carbon and MBC, the non-fumigated sample contained dissolved organic carbon. Soils were frozen prior to DOC and MBC extraction, which may have resulted in cell lysis for both the DOC and MBC extracts leading to an overall reduction in estimated MBC.

### Enzyme assays

Enzyme assays were performed on samples from all plots at each collection date. Each sample was assayed for the potential activity of six different hydrolytic enzymes involved in C, N, and P acquisition (Table 1). The assay protocol was modified from (Saiya-Cork et al., 2002) to include a standard curve for each sample and to minimize quenching effects. The assays for 12 soil samples were incubated for 3 h at 25°C using one deep-well 96-well plate. Two additional plates were used to create standard curves for each sample at 25°C. The reference standard for the leucine amino peptidase assay was 7-amino-4-methylcoumarin (MUC), and for the remaining substrates it was 4-methylumbelliferone (MUB). The standard curve plates had a column for each of the 12 samples and different concentrations of MUB or MUC standards in each well, 0, 2.5, 5, 10, 25, 50, and 100 μM.

**Table 1:** **Enzymes assayed in this study, their abbreviations used in the text, nutrient cycles they are involved in, and their target substrates**.

**Enzyme name**	**Abbreviation**	**Nutrient cycle**	**Enzyme function**
β-glucosidase	BG	C	Hydrolysis of terminal β-D-glucosyl residues
Cellobiohydrolase	CB	C	Hydrolysis of β-D-glucosyl linkages
Xylosidase	XYL	C	Hydrolysis of β-D-xylose residues
Acid phosphomonoesterase	PHOS	P	Hydrolysis of phosphate monoester
N-acetyl glucosaminidase	NAG	N	Hydrolysis of chitin N-acetyl-β-D-glucosaminide
Leucine-amino peptidase	LAP	N	Hydrolysis of N-terminus amino acid leucine

After soils were removed from the freezer, a 2.75 g subsample was taken and warmed to about 20°C. The subsample was homogenized with 91 mL of 50 mM sodium acetate (pH 5.5) for 1 min on high in a Waring blender. Each column on the deep-well 96-well plates corresponded to one sample. After homogenization, 800 μl of soil slurry was aliquoted into six wells of a column on all three plates, one assay plate and two standard plates. Following addition of twelve samples into their respective columns the MUB substrates were added. Each substrate was added to one well in each column, so that all twelve samples received each of the six substrates once.

The plates were incubated for 3 h at 25°C and then centrifuged for 3 min at 350 × g. Afterwards, 250 μl of supernatant from each well was placed into the corresponding well on a 96-well black plate. Fluorescence was measured immediately following 5 μl addition of NaOH to each well to terminate the reaction. A Tecan Infinite M500 spectrofluorometer was used to measure fluorescence with wavelengths set at 365 nm and 450 nm for excitation and emission, respectively. The plates with the standards were used to calculate a linear standard curve and determine potential enzyme activity for each sample as nmol g^−1^ dry soil h^−1^ and nmol g^−1^ C h^−1^.

### Calculations and statistical analysis

Mass-specific enzyme activity was calculated by dividing the potential enzyme activity by the MBC estimated from CFE (Hassett and Zak, 2005). There was no calculation of mass-specific enzyme activity for June 2008 samples because we did not have enough soil to estimate MBC. We included June 2008 samples for all other analyses because it was our pre-warming treatment time point. Ratios for C and N cycling enzymes were calculated as BG:(NAG+LAP) and C:P cycling enzyme ratios as BG:PHOS using potential activity for each sample in nmol g^−1^ C h^−1^ (Sinsabaugh et al., 2009). The ratio of potential activity for different enzymes is a metric for understanding microbial nutrient demand.

Potential enzyme activities were log transformed in order to normalize the variance prior to analysis using SAS PROC GLIMMIX with Tukey's adjustment, α = 0.05 (SAS Institute, Cary, NC). Block and season were selected as random effects, depth, temperature and precipitation treatments were selected as fixed effects and potential enzyme activities, mass-specific enzyme activities, and enzyme stoichiometric ratios were designated as dependent variables. PROC GLIMMIX was used to determine significant field treatment effects within each season and to identify differences among treatments, seasons or depth. MBC estimates were compared within each season using Tukey's comparisons for all treatments.

## Results

### Experimental climate effects

**Figure 1:**
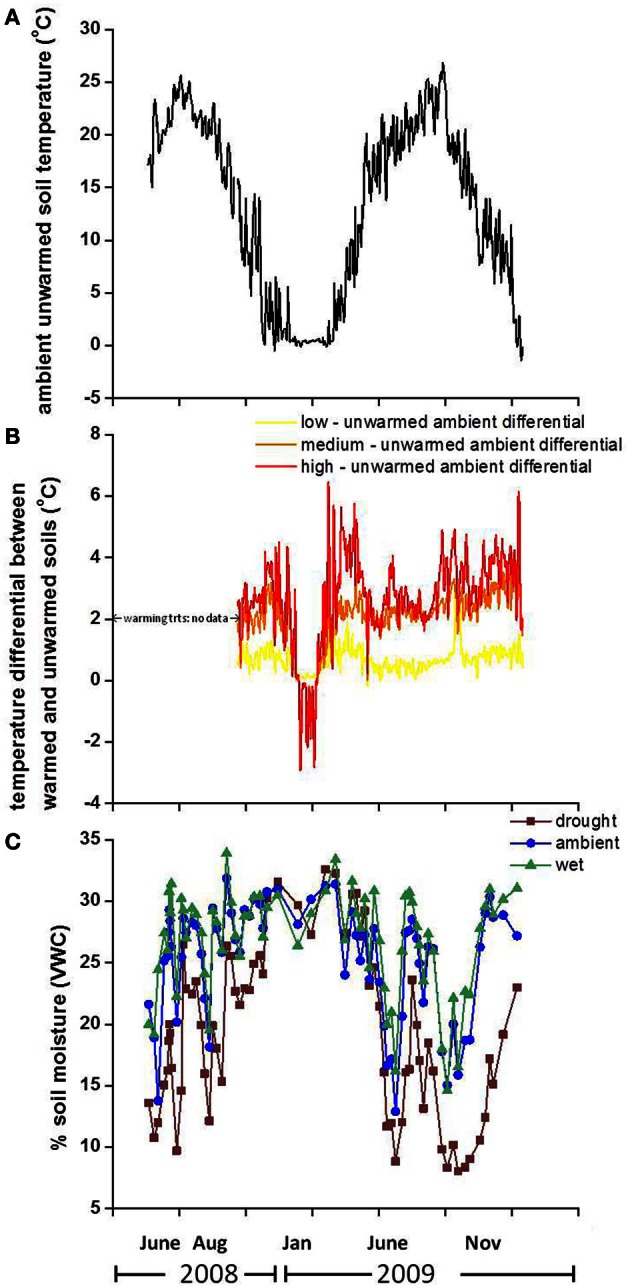
**Soil temperature in the ambient precipitation, unwarmed treatment in 2008 and 2009 (A), the temperature differential between warmed and unwarmed soils (B) and % volumetric soil moisture in 2008 and 2009 (C) by precipitation treatment.** The displayed soil temperature values are from 2 cm below the surface and the moisture panel depicts moisture in the top 10 cm of soil.

Warming treatments increased the soil temperature on average by 0.70, 2.05, and 2.70°C above unwarmed soil temperatures at 2 cm below the surface in both years for the low, medium and high treatments, respectively (Figures 1A,B). There were no soil temperature data available for treatment plots in August 2008. In January 2009, immediately preceding sampling, soil temperatures were 0.37, 1.02, and 1.25°C greater in low, medium, and high temperature plots, respectively, compared to unwarmed plots. However, following the January 2009 sampling, the medium and high temperature treatments soils were cooler than unwarmed soils for the remainder of the month. This counterintuitive pattern resulted in decreased snowpack; by clearing the snow, the warming treatments exposed the soils to freezing air temperature and cooled the soils compared to the unwarmed treatment. Preceding sampling in June 2009, soil temperature increased by 0.43, 2.2, and 2.9°C in low, medium, and high temperature treatments, respectively, compared to the unwarmed treatment. Warming increased the soil temperature in drought and ambient precipitation plots, with the largest soil warming occurring in the drought + high temperature treatment, where soils were 4.0 and 3.5°C warmer than the “unwarmed, ambient” treatment soils during the growing season in 2008 and 2009, respectively.

Precipitation treatments altered soil moisture substantially and soil moisture varied by month at 0–10 cm. Drought treatments resulted in the largest drop in soil moisture during the growing season (Figure 1C). Immediately preceding sampling the soil moisture in drought plots was 20 and 45% lower than ambient soil moisture on the August 2008 and June 2009 sampling dates, respectively. There was no difference in soil moisture among precipitation treatments immediately prior to the January 2009 sampling date. The additional water treatment had no effect on the soil moisture of wet plots in these shallow soil layers, which were already at field capacity in this freely draining, well-structured soil. Soil moisture was further reduced by warming in the drought and ambient precipitation plots in 2008 and 2009.

There was no measurable change in total soil C, N, or C:N ratio due to treatment or seasonal effects. The average total soil C and N values were 57 ± 2.03 and 4.7 ± 0.14 mg g^−1^ dry soil, respectively, for 0–5 cm, and 42 ± 0.67 and 3.6 ± 0.09 mg g dry soil^−1^ for 5–15 cm below the soil surface. Dissolved organic carbon (DOC) was greater in the drought treatments (365.8 ± 25.5) compared to ambient or wet precipitation treatments (217 ± 7.8 and 247 ± 18.11, respectively) at 0–5 cm in June 2009, there was no difference in DOC between treatments on other sampling dates.

### Potential enzyme activities

In all treatments, PHOS and BG had the highest potential activities, and the remaining enzymes exhibited similar activities, usually under 200 nmol activity g^−1^ dry soil h^−1^ (Table 2). At 0–5 cm, warming manipulations individually did not affect potential enzyme activity when calculated per g dry soil. Precipitation treatments also had little overall effect on enzyme activities, but there was a trend toward increased potential activity in drought only plots in June 2009, which was significant for PHOS at 0–5 cm (*P* < 0.05). There was a significant interaction of precipitation × warming treatments on LAP in January 2009 and CB in June 2009 (*P* < 0.05), which always resulted in decreased activity in drought and warmed plots relative to ambient, unwarmed plots.

**Table 2:** **Potential enzyme activity in 0–5 cm soils, mean ± SE**.

**Date**	**Treatment**	**β-glucosidase**	**Cellobiohydrolase**	**Xylosidase**	**Phosphatase**	**N-acetyl glucoaminidase**	**Leucine-amino peptidase**
Jun-08	A + U	320 ± 82	121 ± 27	73 ± 19	65 ± 132	145 ± 43	196 ± 28
	D + U	323 ± 45	120 ± 17	99 ± 18	740 ± 86	350 ± 79	173 ± 18
	W + U	347 ± 90	138 ± 52	79 ± 12	667 ± 102	236 ± 55	169 ± 26
	A + L	309 ± 92	119 ± 31	61 ± 10	617 ± 128	101 ± 26	174 ± 33
	A + M	410 ± 138	171 ± 57	89 ± 27	715 ± 184	204 ± 63	184 ± 45
	A + H	244 ± 45	101 ± 19	44 ± 15	474 ± 92	111 ± 29	164 ± 27
	D + L	219 ± 21	88 ± 12	61 ± 8	497 ± 49	172 ± 5	153 ± 26
	D + M	283 ± 91	110 ± 30	80 ± 28	572 ± 145	189 ± 58	157 ± 13
	D + H	286 ± 72	108 ± 21	69 ± 13	626 ± 150	182 ± 60	162 ± 48
	W + L	281 ± 30	106 ± 9	75 ± 8	652 ± 56	180 ± 25	162 ± 13
	W + M	345 ± 103	137 ± 44	81 ± 18	746 ± 120	229 ± 45	163 ± 4
	W + H	269 ± 35	110 ± 12	65 ± 7	623 ± 74	146 ± 20	172 ± 9
Aug-08	A + U	176 ± 75	96 ± 11	158 ± 109	277 ± 125	118 ± 1	34 ± 19
	D + U	400 ± 176	174 ± 84	152 ± 58	578 ± 384	301 ± 119	70 ± 14
	W + U	235 ± 60	158 ± 53	75 ± 2	479 ± 68	187 ± 33	74 ± 18
	A + L	250 ± 34	96 ± 9	56 ± 3	416 ± 71	101 ± 17	55 ± 24
	A + M	188 ± 91	426 ± 362	235 ± 174	298 ± 148	262 ± 167	41 ± 9
	A + H	339 ± 133	178 ± 65	170 ± 106	1159 ± 748	686 ± 567	57 ± 17
	D + L	122 ± 60	88 ± ^*^	151 ± 59	226 ± 129	181 ± ^*^	48 ± 16
	D + M	250 ± 108	112 ± 72	124 ± 79	430 ± 172	158 ± 65	49 ± 9
	D + H	1031 ± 933	57 ± 30	68 ± 36	178 ± 51	66 ± 19	37 ± 12
	W + L	278 ± 23	252 ± 73	147 ± 46	506 ± 56	335 ± 103	51 ± 12
	W + M	169 ± 76	173 ± 41	123 ± 41	273 ± 130	248 ± 100	84 ± 9
	W + H	198 ± 54	66 ± 44	70 ± 33	303 ± 120	77 ± 38	56 ± 14
Jan-09	A + U	820 ± 221	265 ± 88	144 ± 50	1588 ± 542	386 ± 100	120 ± 38
	D + U	275 ± 62	144 ± 18	134 ± 26	1083 ± 158	240 ± 25	134 ± 38
	W + U	1067 ± 302	410 ± 124	263 ± 83	2352 ± 566	521 ± 152	140 ± 41
	A + L	501 ± 143	178 ± 47	84 ± 18	973 ± 181	219 ± 60	53 ± 7
	A + M	800 ± 204	308 ± 84	164 ± 38	1537 ± 511	301 ± 57	93 ± 16
	A + H	687 ± 315	264 ± 125	114 ± 45	1234 ± 443	249 ± 125	93 ± 52
	D + L	365 ± 35	137 ± 3	88 ± 16	799 ± 124	156 ± 29	92 ± 29
	D + M	549 ± 254	210 ± 104	114 ± 20	998 ± 344	244 ± 76	85 ± 21
	D + H	273 ± 13	98 ± 2	56 ± 6	543 ± 36	119 ± 11	42 ± 9
	W + L	506 ± 54	200 ± 31	133 ± 37	1227 ± 176	212 ± 32	72 ± 10
	W + M	549 ± 109	228 ± 59	182 ± 54	1736 ± 612	391 ± 133	106 ± 40
	W + H	699 ± 372	276 ± 168	165 ± 99	1673 ± 851	310 ± 171	60 ± 44
Jun-09	A + U	226 ± 27	82 ± 19	58 ± 9	416 ± 63	117 ± 22	96 ± 54
	D + U	341 ± 44	150 ± 39	130 ± 58	647 ± 701	264 ± 78	154 ± 63
	W + U	264 ± 17	101 ± 10	77 ± 13	474 ± 46	118 ± 18	74 ± 31
	A + L	174 ± 31	66 ± 13	38 ± 6	262 ± 71	68 ± 18	95 ± 56
	A + M	223 ± 29	99 ± 11	66 ± 12	411 ± 30	87 ± 10	76 ± 21
	A + H	234 ± 32	95 ± 13	45 ± 9	371 ± 48	106 ± 35	69 ± 28
	D + L	291 ± 48	125 ± 32	86 ± 25	496 ± 99	180 ± 51	168 ± 52
	D + M	232 ± 51	78 ± 16	46 ± 7	307 ± 28	111 ± 19	121 ± 47
	D + H	261 ± 59	95 ± 26	74 ± 24	368 ± 83	155 ± 24	130 ± 54
	W + L	526 ± 252	104 ± 16	51 ± 25	410 ± 108	93 ± 48	79 ± 24
	W + M	217 ± 40	88 ± 20	58 ± 14	400 ± 32	100 ± 20	111 ± 47
	W + H	364 ± 102	166 ± 44	122 ± 36	639 ± 140	203 ± 73	166 ± 37

Treatment abbreviations are noted by precipitation and temperature manipulations, A, ambient precipitation; D, drought; W, 150% ambient precipitation; U, unwarmed; L, low warming; M, medium warming; H, high warming.

* n = 1.

In deeper soils (5–15 cm), warming alone tended to decrease potential activity for all enzymes in the medium-warmed plots (data not shown; *P* = 0.2) compared to the “unwarmed, ambient” plots, and this effect was significant for XYL and LAP (*P* < 0.01). Potential enzyme activities were significantly lower in January 2009 at 5–15 cm below the surface for NAG, XYL, and LAP compared to August 2008 (data not shown; *P* < 0.05).

### Microbial biomass

**Figure 2:**
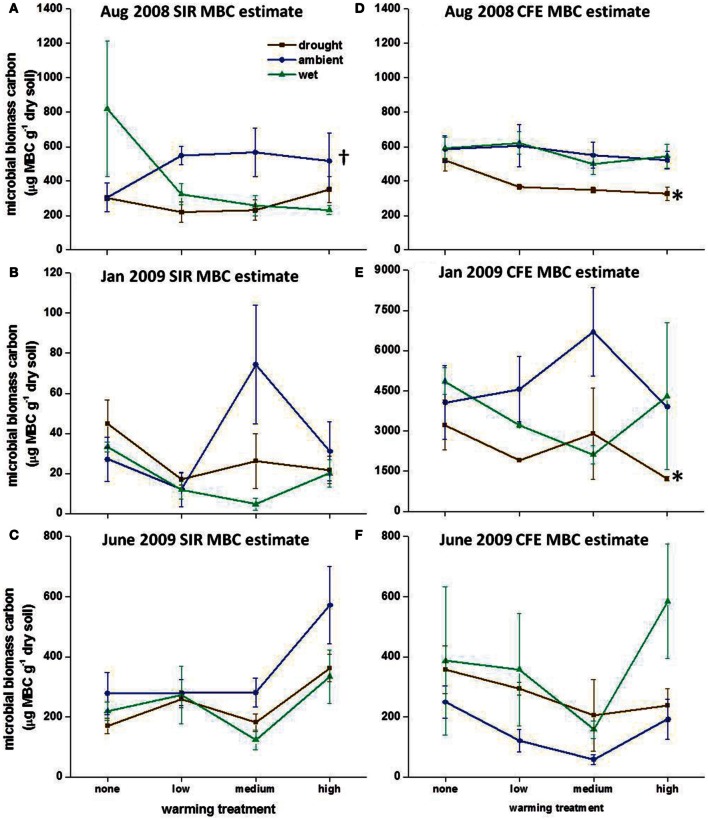
**Microbial biomass carbon (MBC) calculated using substrate-induced respiration (SIR) in (A) August 2008, (B) January 2009, and (C) June 2009 and using chloroform fumigation extraction (CFE) in (D) August 2008, (E) January 2009, and (F) June 2009.** Symbols show averages with standard error bars, *n* = 3. Crosses indicate significant differences in MBC between temperature treatments under ambient precipitation and asterisks indicate a significant difference in MBC between precipitation treatments without warming (*P* < 0.05). Note different y-axes in January 2009 for SIR and CFE estimated MBC.

In August 2008, warming alone resulted in slightly higher SIR-MBC estimates compared to the “unwarmed ambient” plots in August 2008, but in the drought and wet treatments warming had no effect on SIR-MBC (Figure 2A). In January 2009 there was no consistent effect of individual or combined field treatments on SIR-MBC (Figure 2B). CFE-MBC was affected by precipitation in August 2008 and January 2009, but there was no effect of either climate manipulation in June 2009 (Figures 2D–F). Although SIR-MBC and CFE-MBC were similar in August 2008 and June 2009, SIR was lower than CFE in January 2009 (Figure 2; *P* < 0.005).

### Mass specific enzyme activity

**Figure 3:**
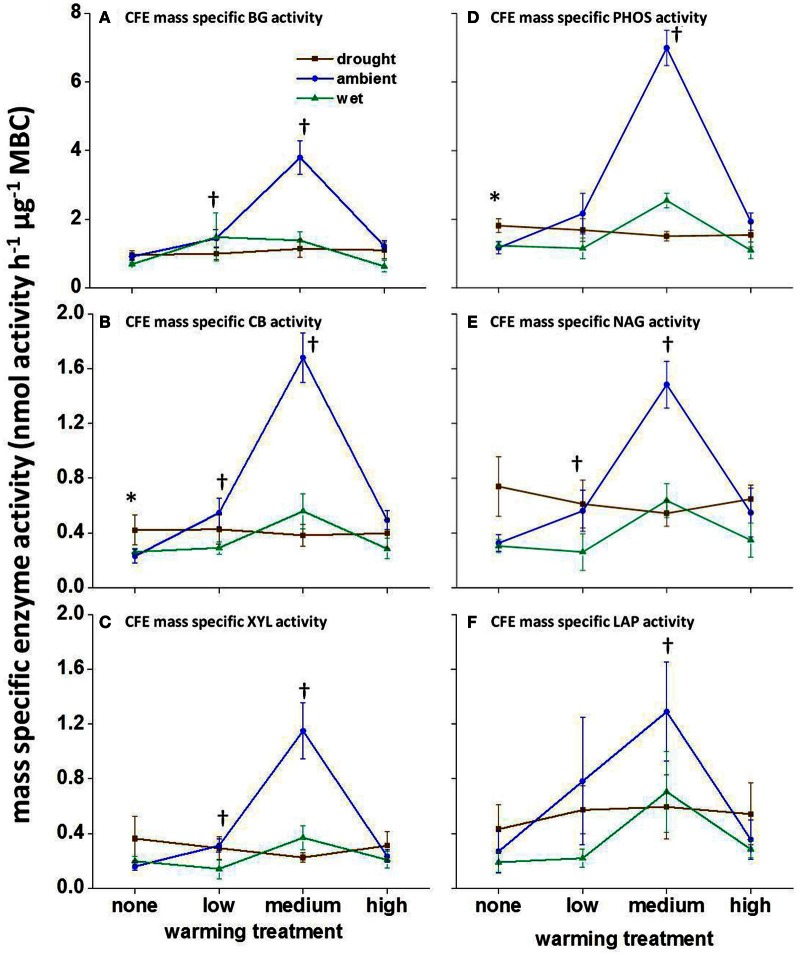
**Mass-specific potential enzyme activity in June 2009 calculated using CFE estimated biomass.** CFE estimated mass specific enzyme activity for **(A)** BG, **(B)** CB, **(C)** XYL, **(D)** PHOS, **(E)** NAG, and **(F)** LAP. For each substrate, asterisks indicate significant differences in mass-specific enzyme activity (*P* < 0.05) between the precipitation treatments without warming. Crosses indicate significant differences in mass-specific enzyme activity (*P* < 0.01) between temperature treatments under ambient precipitation. Substrate abbreviations are noted in Table 1. Averages and standard errors, *n* = 3. Note different y-axis scales in panels.

The climate manipulations did not affect mass-specific enzyme activity in August 2008 or January 2009 (nmol activity h^−1^ μ g^−1^ MBC; calculated using CFE-MBC estimates; data not shown). In June 2009, the mass specific enzyme activity for all enzymes was affected by warming alone, with mass-specific enzyme activity increasing under low- and medium-warmed treatments (Figure 3; *P* < 0.01). Additionally, in June 2009 precipitation had a significant effect on PHOS and CB, with drought only plots having higher mass specific enzyme activity than wet and ambient precipitation plots (Figures 3B,D; *P* < 0.05).

### Enzyme stoichiometry

**Figure 4:**
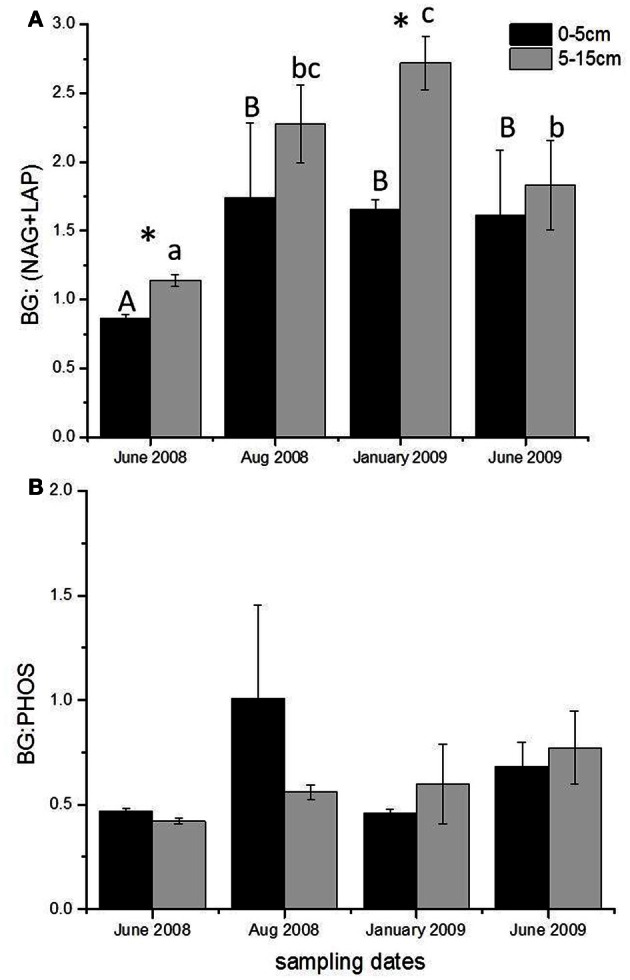
**Stoichiometric ratios of potential enzyme activity at different sampling dates for both depths, **(A)** BG: (NAG + LAP) at 0–5 and 5–15 cm depths and **(B)** BG: PHOS at 0–5 and 5–15 cm depths below the soil surface.** Different capital letters and lower case letters, for 0–5 cm and 5–15 cm depths respectively, indicate significant differences between sampling dates, and an asterisk indicates a significant difference by depth (*P* < 0.05). Averages and standard errors were calculated using all the plots for each date due to the lack of treatment effect on enzyme stoichiometry; *n* = 36.

Season affected the ratio of the potential activities of C- to N-acquiring enzymes at 5–15 cm below the surface, with a significant increase in the ratio in winter 2009 compared to the two June samples (*P* < 0.01, Figure 4A). The C:N enzyme activity ratio increased from June 2008 to January 2009 and then declined in June 2009, whereas C- to P-acquiring enzyme ratios showed no seasonality (Figure 4B). There was also a significant depth effect in the C:N enzyme activity ratio for June 2008 and January 2009, with soils from the 5–15 cm depth having a higher ratio than those from 0 to 5 cm (*P* < 0.05).

## Discussion

### Precipitation and warming effects

Previous studies of climate change effects on enzyme activities, decomposition, and heterotrophic respiration have focused primarily on warming effects. Climate effects on soil enzyme activities involve not only short-term changes in activity driven by thermodynamics (Trasar-Cepeda et al., 2007) but also long-term changes in enzyme pools due to direct and indirect effects on microbial production of enzymes and on turnover rates (Sowerby et al., 2005; Schimel et al., 2007). A recent study pointed out that there is an even greater uncertainty associated with the effects of altered soil moisture [through both warming and altered precipitation patterns (Falloon and Betts, 2010)]. Here, we assessed the interactive effects of warming and precipitation on soil enzymes relative to MBC.

We predicted that potential enzyme activities would decrease in response to drought due to decreased microbial biomass and enzyme production. However, we observed no change in the potential activities of any of the enzymes involved in C and N cycling in any precipitation treatment, and a small increase in phosphatase under drought, whereas both total microbial biomass and field respiration declined under drought (Suseela et al., 2012). Several other studies in oxic soils have detected declines in hydrolytic and oxidative potential enzyme activities under drought conditions (Sardans et al., 2008; Toberman et al., 2008; Sardans and Penuelas, 2010). Most of these studies have been in Mediterranean systems, which are drought-prone, whereas drought is a less frequent condition at the BACE location. The stable enzyme pool under drought could indicate that either mass-specific enzyme production was higher under drought, or more likely that enzyme turnover decreased in dry soils.

Although we measured stable potential enzyme activities under drought, this assay does not necessarily indicate that these enzymes were active *in situ* in dry soils. Suseela et al. (2012) measured a 21% reduction in heterotrophic respiration under drought at BACE, suggesting that decomposition rates are lower in drought plots despite potential enzyme activities similar to ambient conditions. This may be due either to reduced enzyme activities in dry soils or because of reduced microbial uptake of assimilable DOM. Potential enzyme activity as measured in laboratory assays does not necessarily directly correlate with *in situ* activity under field conditions (Wallenstein and Weintraub, 2008). Using a combination of empirical data and modeling, we previously determined that *in situ* enzyme activity at the BACE is significantly reduced by drought, such that despite a stable pool of enzymes, the actual activity is constrained by a lack of moisture and diffusion (Steinweg et al., 2012). Under low soil moisture conditions, the diffusion of enzymes and substrates is limited to thin water films and pockets of moisture with low connectivity (Stark and Firestone, 1995). As substrates are concentrated in these hotspots, enzyme activities could continue even in relatively dry soils, resulting in the production of assimilable DOM. The accumulation of DOM under drought would indicate that microbial uptake is more sensitive to soil moisture than enzyme activity. Consistent with this mechanism, we observed higher concentrations of DOC in drought plots compared to ambient or wet plots in June 2009, when drought treatment soils were 50% drier than ambient and wet soils. If enzyme activities persist in dry soils but microbial uptake is suppressed, declines in microbial respiration would mask the continuation of decomposition in dry soils. This mechanism could also explain the pulse of respiration that often accompanies rewetting in laboratory incubations and field studies (Fierer and Schimel, 2003; Schimel et al., 2007), as accumulated DOM is rapidly metabolized by microbes upon rewetting.

Warming increased mass-specific potential enzyme activity under low- and medium-warmed treatments, such that more enzymes were present per unit of MBC in June 2009. Enzyme reactions are temperature-sensitive, and we had expected that *in situ* enzymatic reaction rates would increase with field warming, reducing the number of enzymes needed to perform the same number of reactions. However, warming not only affects extracellular enzyme reaction rates, but also affects the reactions occurring within the microbial cell. Maintenance costs also increase with temperature (Joergensen et al., 1990; Alvarez et al., 1995), causing an increased nutrient demand to maintain cellular function. Several models suggest that microorganisms increase allocation of nutrients to enzyme production in order to acquire the nutrients needed to sustain increased maintenance costs with warming (Schimel and Weintraub, 2003; Wang and Post, 2012; Wang et al., 2013).

### Seasonal trends

SIR-MBC and CFE-MBC were similar during the growing season. However, in winter SIR was lower than CFE. SIR-MBC is often interpreted to indicate the size of the active microbial biomass pool whereas CFE-MBC indicates the total microbial biomass pool (Wardle and Parkinson, 1991; Lipson et al., 1999). During the growing season it appears that the total microbial community was active, whereas in winter a very small subset was active at the BACE site. The increase in CFE-MBC during winter is similar to the results from Lipson et al. (1999), however, they measured similar increases during winter in their SIR-MBC estimates as well. Our use of a consistent incubation temperature, 25°C, for SIR-MBC may have underestimated MBC during the winter if the community was better adapted to colder temperatures at that time, however, other work indicates that bacterial growth rate in temperate regions are higher than 25°C (Rousk et al., 2012).

The most striking response of enzymes to season was a change in the stoichiometry of potential enzyme acquisition activities. Sinsabaugh et al. (2009) reported an average enzyme C:N acquisition ratio (BG activity: NAG + LAP activities) close to 1.41 for soils from 40 ecosystems. The average enzyme C:N acquisition activity ratio in BACE soils was 1.74, driven primarily by the high stoichiometric ratio at 5–15 cm depth. The increase in enzyme C:N acquisition activity from June 2008 to January 2009 was driven by both an increase in C-acquiring enzymes and a decrease in the potential activity of N-acquiring enzymes in the winter. Maintenance costs continue and may increase with freezing events (Methe et al., 2005), resulting in a continual need for C substrates, without a corresponding increase in N demand. In addition, increased C mineralization:N mineralization was measured during winter at the BACE site (Auyeung et al., 2012), indicating increased microbial C utilization relative to N transformation. The decline in potential activity of N-acquiring enzymes in winter relative to C-acquiring enzymes indicated a reduction in organic N degradation in the winter compared to the growing season. The reduction in organic N acquiring enzymes could possibly be due to increased dissolved N (Chróst, 1991), which was measured in the winter at the BACE (data not shown). The average BG:PHOS ratios at the BACE, 0.73, were similar to the reported average of 0.62 for soils (Sinsabaugh et al., 2009). The stability of enzymatic C:P activity ratios through time suggests a consistent P requirement over the year. Even though there may be consistent potential enzyme activities in the winter and summer for some enzymes, it is unlikely that *in situ* activity is the same (Bell et al., 2010). Low soil temperatures would result in slower reaction rates and frozen soils would limit diffusion of substrates resulting in reduced *in situ* activities.

## Conclusion

Our findings from 1 year of climate manipulations suggest that neither experimental warming nor moisture manipulation consistently affected potential enzyme activities. The stable enzyme pool under drought could indicate that either mass-specific enzyme production was higher under drought, or more likely that enzyme turnover decreased in dry soils. Experimental warming did impact mass-specific enzyme activities through small decreases in MBC and small increases in potential enzyme activity, indicating increased allocation to enzyme production. Seasonal shifts in C:N acquisition enzyme activity ratios, resulting in increased potential for acquisition for processing C during the winter, could be due to increased maintenance costs associated with freezing events. The shifts in mass-specific enzyme activity and enzyme stoichiometry indicate increased microbial allocation to enzymes during periods when maintenance costs were likely to be high due to high temperatures, similar to results predicted in microbial enzyme-mediated models (Schimel and Weintraub, 2003; Wang et al., 2013). Our results highlight the need to elucidate how abiotic and biotic factors affect the relationship between maintenance costs and enzyme activities to better predict microbial responses to future climate regimes.

### Conflict of interest statement

The authors declare that the research was conducted in the absence of any commercial or financial relationships that could be construed as a potential conflict of interest.
